# The enigmatic Otway odd-clawed spider (*Progradungula otwayensis* Milledge, 1997, Gradungulidae, Araneae): Natural history, first description of the female and micro-computed tomography of the male palpal organ

**DOI:** 10.3897/zookeys.335.6030

**Published:** 2013-09-25

**Authors:** Peter Michalik, Luis Piacentini, Elisabeth Lipke, Martin J. Ramírez

**Affiliations:** 1 Allgemeine und Systematische Zoologie, Zoologisches Institut und Museum, Ernst-Moritz-Arndt-Universität, J.-S.-Bach-Str. 11/12, D-17489 Greifswald, Germany; 2 Research Associate, Division of Invertebrate Zoology, American Museum of Natural History, Central Park West at 79th Street, New York, NY 10024, USA; 3 Museo Argentino de Ciencias Naturales - CONICET, Buenos Aires, Argentina

**Keywords:** Taxonomy, micro-CT, palp, spermatheca, web, *Nothofagus*

## Abstract

The recently described cribellate gradungulid *Progradungula otwayensis* Milledge, 1997 is endemic to the Great Otway National Park (Victoria, Australia) and known from only one male and a few juvenile specimens. In a recent survey we recorded 47 specimens at several localities across the western part of the Great Otway National park. Our field data suggest that this species is dependant on the microclimate in the hollows of old myrtle beech trees since other hollow trees were very much less inhabited. Furthermore, we describe the female for the first time and study the male palpal organ by using X-ray microtomography. The female genitalia are characterized by eight spermathecae which are grouped in two quartets. The spermophor resembles the general organization of gradungulids, but is similar to *Kaiya* Gray, 1987 by a convoluted appearance within the embolus. The muscle 30 is located in the cymbium and resembles the organization of other non-entelegyne Araneomorphae.

## Introduction

The family Gradungulidae consists of seven genera with 16 described species from eastern Australia and New Zealand (Platnick 2013). The genera *Macrogradungula* (*Macrogradungula moonya* Gray, 1987) and *Progradungula* (*Progradungula carraiensis* Forster and Gray, 1979 and *Progradungula otwayensis* Milledge, 1997) can be distinguished from the remaining gradungulids by being cribellate. These spiders use a very conspicuous catching ladder for prey capture (Forster et al. 1987; Gray 1983; Figs 2B–C). The enigmatic *Progradungula carraiensis* is known only from the Carrai Bat Cave in northern New South Wales (Forster and Gray 1979; Gray 1983) and thus it was remarkable when Milledge (1997) described a second species, *Progradungula otwayensis*, from the Great Otway National Park in southern Victoria (Australia). Nevertheless, only a single adult male and several immatures were reported at that time. In the present paper, we describe the female for the first time and reconstruct the male palpal organ by using X-ray microtomography (micro-CT). Moreover, we provide new data on the natural history of this species.

## Material and methods

**Figure 1.**
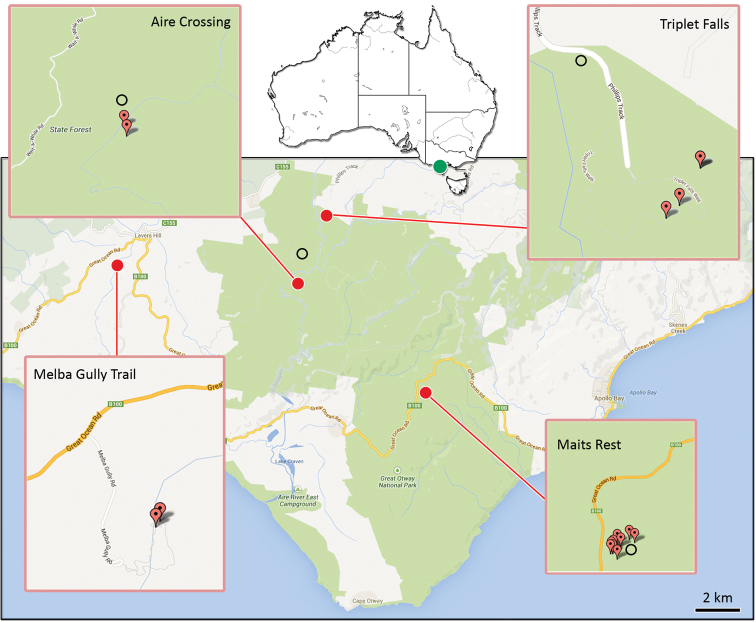
Distribution map of *Progradungula otwayensis* in the Great Otway National Park, Victoria (Australia). Red marks indicate localities for the present study; black circles indicate localities given by Milledge (1997).

We observed a total of 47 specimens (juveniles, females, one male) in the localities depicted in Fig. 1. Several individuals were collected for further analyses. The female genitalia were digested following the protocol of Álvarez-Padilla and Hormiga (2007). The material was examined and documented (extended focal range images) in 80% ethanol using a Zeiss Discovery V20 stereo microscope with a Zeiss MCr camera and a Leica M205A with a Leica 290 camera. Editing of images to adjust brightness, contrast and color was performed using Adobe Photoshop CS4. The measurements and description are based on Forster et al. (1987), the description of the spination follows Ramirez (2003).

**Figure 2.**
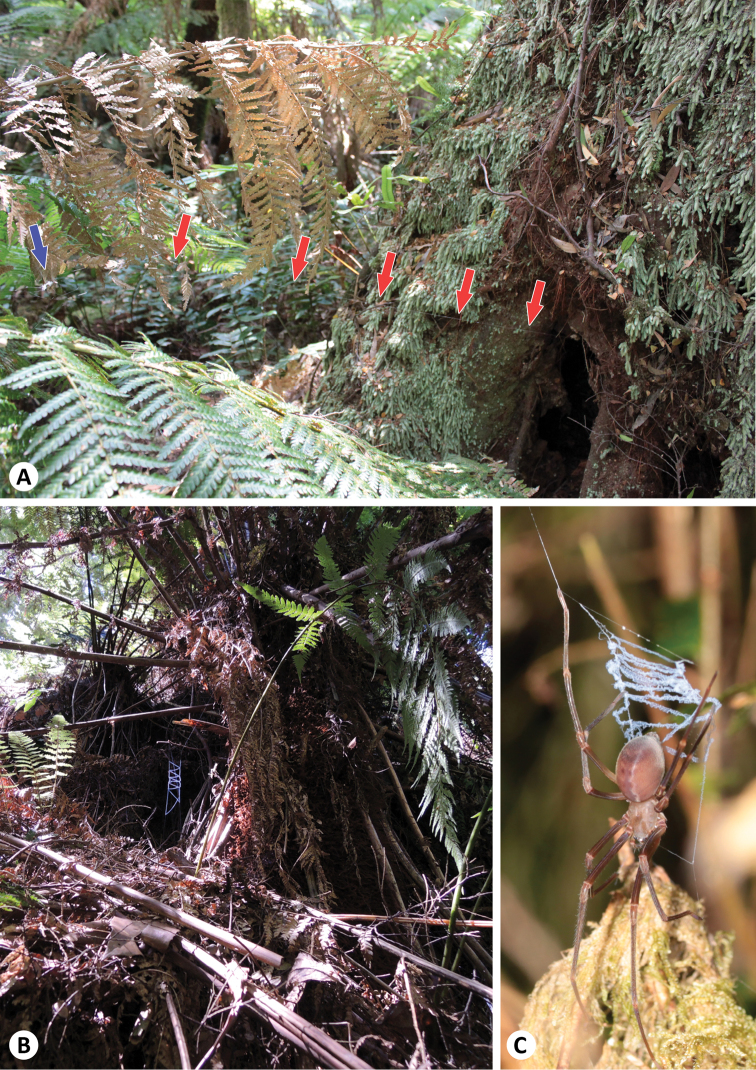
**A** Habitat with supporting web (blue arrow) and sturdy thread (red arrows) connecting with the retreat in the hollow of a *Nothofagus cunninghamii* tree **B** Catching ladder in front of a hollow in a large tree fern **C** Immature male of *Progradungula otwayensis* holding the catching ladder.

For the micro-CT analyses of the male palp, the sample was dehydrated in graded ethanol and stained with a 1% iodine solution for 12 hours. After washing in pure ethanol, the sample was scanned in ethanol with an Xradia MicroXCT-200 X-ray imaging system (Carl Zeiss X-ray Microscopy Inc., Pleasanton, USA) at 20 KV and 4 W (10.0 scintillator-objective lens unit, 11 s exposure time, 2.18 µm pixel size). The female genitalia were digested with enzymatic cleaner for contact lenses Ultrazyme, and dried after the iodine treatment using hexamethyldisilazane (HMDS). The scan was performed using the same system at 30 kV and 6 W (20.0 scintillator-objective lens unit, 6 s exposure time, 1.18 µm pixel size). The obtained data were processed using the 3D analysis software AMIRA v. 5.4.2 (Visage Imaging, Berlin, Germany). The spermophor was reconstructed by delineation of the contours in each section and a smooth surface was computed using the surface editor. The image stack is stored in MorphDBase under creative commons attribution (ID: P_Michalik_20130802-M-5.1, https://www.morphdbase.de/?P_Michalik_20130802-M-5.1).

### Abbreviations

ALE anterior lateral eyes

AME anterior median eyes

B bursa

bH basal hematodocha

Cb cymbium

E embolus

EF epigastric furrow

Gl glands

GP genital pockets

m29 muscle 29

m30 muscle 30

mA median apophysis

mH median hematodocha

PE process of embolus

PF postepigastric fold

PLE posterior lateral eyes

PME posterior median eyes

S spermophor

Sp spermatheca

St subtegulum

Te tegulum

Ue uterus externus

tm29 tendon of muscle 29

tm30 tendon of muscle 30

MACN Museo Argentino de Ciencias Naturales (Buenos Aires, Argentina)

MV Museum of Victoria (Melbourne, Victoria)

ZIMG Zoologisches Institut und Museum Greifswald (Germany)

## Taxonomy

### Family Gradungulidae Forster, 1955
Genus *Progradungula* Forster & Gray, 1979

#### Progradungula otwayensis

Milledge, 1997

http://species-id.net/wiki/Progradungula_otwayensis

##### Type material.

Male holotype: AUSTRALIA: Victoria. Otway Ranges, Aire Crossing Track, 0.5 km N of Aire River crossing, 38°42’S, 143°29’E, 31 Jan 1995, G. Milledge (MV K3260, not examined).

##### Material examined.

AUSTRALIA: VICTORIA: Great Otway National Park: Little Aire Cascade Trail, E of Lavers Hill, 38.67032°S, 143.49810°E (GPS, ±100m), elev. 330m, 11 Feb 2013, *Nothofagus*, *Eucalyptus*, tree ferns wet forest, hand collecting, P.Michalik & M.J.Ramírez (MJR-Loc-125), two females (MACN-Ar 30666); Triplet Falls Trail, E of Lavers Hill, 38.67188°S, 143.49673°E (GPS, ±300m), elev. 300m (GPS), 10 Feb 2013, *Nothofagus*, *Eucalyptus*, tree ferns wet forest, hand collecting, P.Michalik & M.J.Ramírez (MJR-Loc-124), three females (MACN-Ar 30667, ZIMG II/28128, ZIMG II/28129); Maits Rest Trail, W Apollo Bay, 38.75492°S, 143.55495°E (GPS, ±200m), elev. 240m, 13–14 Feb 2013, *Nothofagus*, tree ferns wet forest, hand collecting, P.Michalik & M.J.Ramírez (MJR-Loc-126), one female (ZIMG II/28130); Melba Gully Trail, 38°41.726’S, 143°22.312’E (GPS, ±200m), elev. 328m, 15 Feb 2013, *Nothofagus*, tree ferns wet forest, hand collecting, P.Michalik & M.J.Ramírez (MJR-Loc-128), one male (ZIMG II/28127).

##### Diagnosis.

This species can be distinguished from *Progradungula carraiensis* by the single process on the embolus of the male palpal organ and the presence of eight spermathecae in the female genitalia.

##### Female

(Figs 3–4).

**Figure 3.**
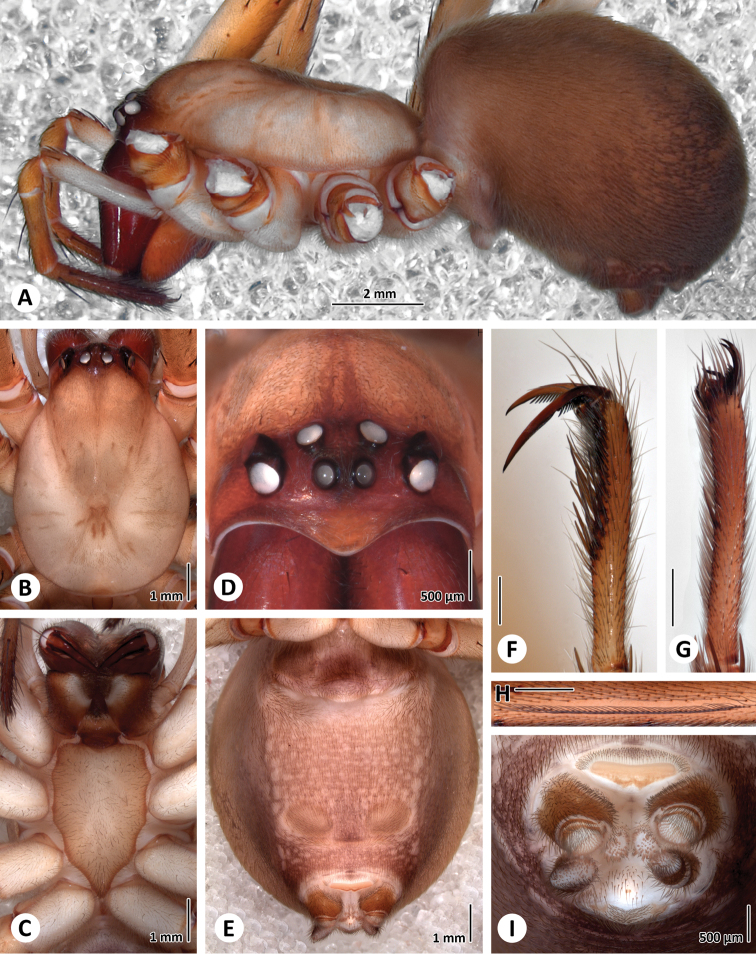
Somatic characters of the female of *Progradungula otwayensis*. **A** Lateral view of prosoma and opisthosoma (ZIMG II/28128) **B** Dorsal view of prosoma (MV) **C** Ventral view of Prosoma (MV) **D** Frontal view of ocular area (ZIMG II/28128) **E** Ventral view of opisthosoma **F** Tarsus of leg I **G** Tarsus of leg IV **H** Calamistrum. **I** Ventral view of spinnerets. Scale bar in **F–H** is 500 µm.

**Figure 4.**
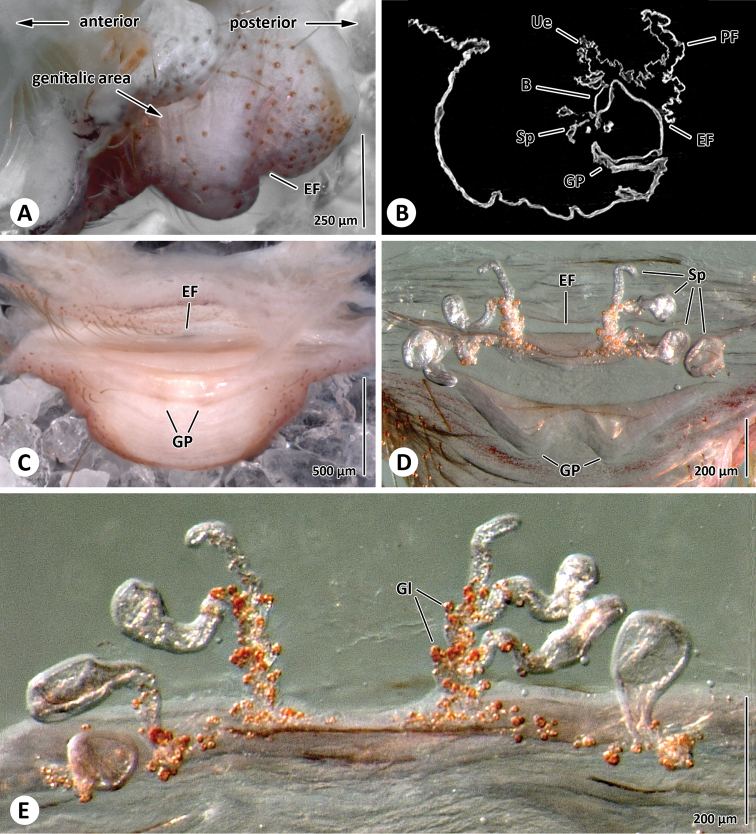
Female genitalia. **A** Lateral view of the dome-shaped genital area (compare also to Fig. 3A) (MACN-Ar 30667) **B** Sagital section through genital area obtained by micro-CT (MACN-Ar 30667) **C** Posterior view of genital area (MACN-Ar 30667). Spermathecae in anterior (**D**) and ventral view (**E**) (ZIMG II/28128). Abbreviation: **B** bursa; **EF** epigastric furrow; **GP** genital pockets; **Gl** glands; **PF** postepigastric fold; **Sp** spermathecae; **Ue** uterus externus.

##### Measurements

(mm, in MV). Carapace length 6.92; carapace width 4.66; caput width 2.66; abdomen length 8.51; abdomen width 7.18. Legs: length of segments (femur + patella/tibia + metatarsus + tarsus = total length): I 11.97 + 14.10 + 11.57 + 2.66 = 40.30, II 9.31 + 10.77 + 8.25 + 2.53 = 30.86, III 7.98 + 9.18 + 7.85 + 2.13 = 27.14, IV 9.98 + 10.91 + 9.44 + 2.13 = 32.46, palp 2.93 + 2.66 + - + 2.79 = 8.38.

##### Colour pattern.

Carapace yellowish brown, darker on cephalic area, particularly in eye region. Chelicerae, maxillae and labium reddish brown. Sternum reddish brown marginally, paler centrally. Legs yellowish brown. Abdomen fawn-coloured with dark brown dorsal pigmentation consisting of 3 chevron markings. Carapace longer than wide, widest between coxae II and III. Cephalic area elevated behind eyes (Fig. 3A). Clypeus sloping, as long as the median ocular quadrangle, with a strongly curved anterior border. Fovea pit-like.

##### Eyes.

Frontal view (Fig. 3D): anterior row slightly recurved, posterior row slightly procurved. Dorsal view (Fig. 3B): anterior row recurved, posterior row slightly recurved. AME < PME < ALE = PLE 0.23: 0.32: 0.35: 0.35. Interdistances: AME-AME 0.18, AME-ALE 0.33, ALE-PLE 0.08, PLE-PME 0.40, PME-PME 0.28. Median ocular quadrangle: length 0.58 mm, anterior width 0.55 mm, posterior width 0.87 mm. Clypeus height 0.50. AME black, remainder white.

##### Chelicerae.

Strong, vertical, slightly divergent. Three strong prolateral teeth, evenly spaced. Five very small retromarginal teeth (or denticles) in row on basal half of groove, with a spine on the apico-dorsal side. Stridulatory ridges absent.

##### Maxillae.

Subparallel, external margin strongly curved and ending anteriorly in a bluntly pointed apex (Fig. 3C). Serrula present.

##### Labium.

Free. Length 0.83, width 0.87. Apical margin indented, lateral margin subparallel below, sloping in toward apex above (Fig. 3C).

##### Sternum.

Length 3.33, width 2.20. Elongate, shield-shaped with pointed apex which extends back midway between coxae IV (Fig. 3C).

##### Legs.

1423. Trochanters shallowly notched. Superior claws of 1st and 2nd legs dissimilar, with raptorial proclaws long and strongly developed (Fig. 3F), retroclaws shorter. Inferior claws of legs I and II slender and strongly hooked. Superior claws of legs III and IV similar (Fig. 3G). Accessory claw setae on all tarsi. Distal half of tarsus I and II with strong ventral setae. Calamistrum short, located in second quarter of metatarsus 4 (Fig. 3H).

##### Pattern of spination.

(Approximate, slightly asymmetrical.) Femur I d (r2p1)-r2-r1-p1-1-2-p1-2-p1-2-2-3ap v 0-p1-0-0-0; patella r 1; tibia d 2-0-2-p1-0-p1-3 v 2-0-0-2-p1-0-2-0; metatarsus d r1-p1-p1-0-0-0-0 v p1-r1-p1-p1-r1-p1-r1-2ap; II; femur d (r2p1)-r2-2-p1-2-p1-2- 2-p1-3 v 0-p1-1-0-0; patella p 1; tibia d 2-p1-1-2-2-p1-p1-1-3 v 2-0-p1-r1-p1-2-0-2ap; metatarsus d 2-p1-2-r1-p1-r1-p1-p1-2ap v p1-r1-2-2-r1-p1-3ap; III; femur d (r3p1)-1-r1-2-p1-2-2-2-r1-3 v r1-p1-2-0-0; patella p 1 d 1 r 1; tibia d 2-1-p1-3-2-2 v 2-0-p1-r1-p1-2ap; metatarsus d 2-p1-p1-r1-p1-2- 2ap v 2-p1-p1-r1-p1-p1-r1-3ap; tarsus v 0-r1-0; IV femur d (r4p2)-r2-1-r2-p1-2-2-2-3 v 0-p1-p1-0-0-0; patella p 1 d 1; tibia d 2-2-p1-1-3-r1 v 2-0-0-p1-p1-2ap; metatarsus d 2-p1-2-r1-2ap v p1-2-p1-p1-p1-2-r1-3ap; tarsus v 0-1-0.

##### Palp.

Single claw. Spines: femur d 0-0-1-1-3ap patella p 1 d 1-1, tibia d 1-1, p 0-2-0; tarsus d 2-2-1-2-3ap v 2-2-2ap.

##### Abdomen.

Cribellum undivided, with narrow spinning field, as wide as basal span of anterior spinnerets (Figs 3E, 3I). Spiracles of posterior lung books well separated but joined by a transverse groove. Genital area swollen and only faintly sclerotized near the epigastric furrow (Fig. 3E).

##### Genitalia.

Genital area distinct and dome-shaped (Figs 3A, 4A). Internal genitalia simple, with eight spermathecae grouped in two quartets (Figs 4D–E). The outer spermathecae are ovoid and have a short duct. The inner spermathecae can be grouped leading into the bursa through a long, slender convoluted duct (Fig. 4). Glandular projections are mainly present on the spermathecal ducts. A fold with two distinct sclerotized pockets is situated anterior/ventral to the spermathecae. A blind-ended invagination, the postepigastric furrow, is situated immediately posterior to the epigastric furrow.

##### Male

**Figure 5.**
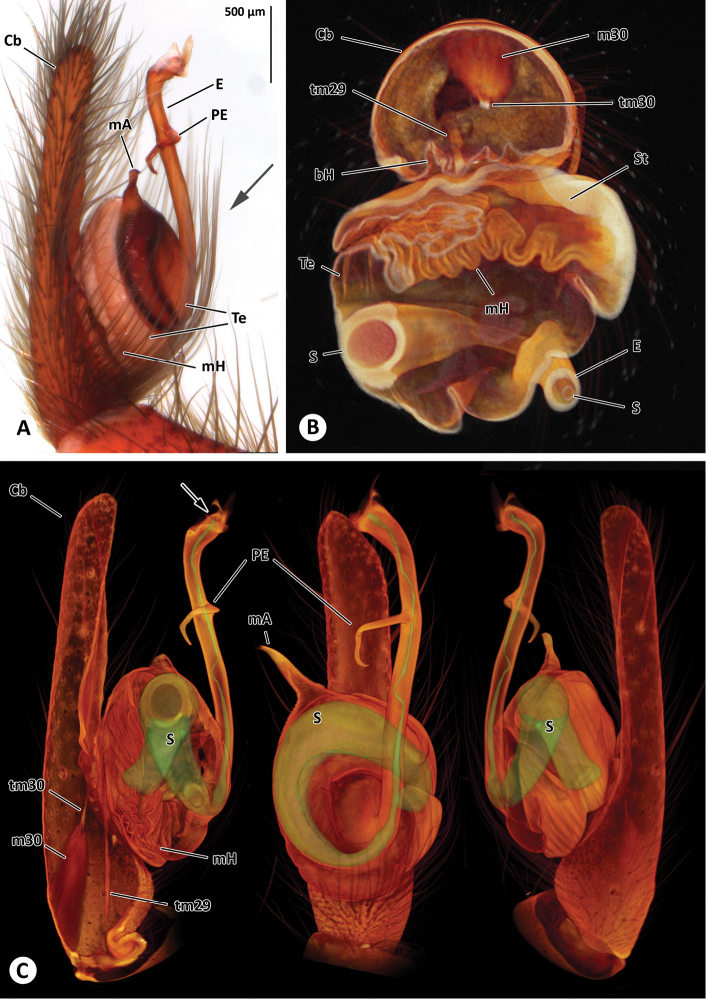
Right male palp of *Progradungula otwayensis* (ZIMG II/28127). **A** Prolateral view; the arrow marks the section plane of Fig. 5B **B** Section of the volume rendered male palp **C** Surface model of the spermophor superimposed on the volume rendering of the male palp (prolateral, ventral and retrolateral views). The cymbium, subtegulum and tegulum are partly removed in the prolateral view to show tendons, muscles and hematodochae. The arrow points to the opening of the embolus. Abbreviation: **Cb** cymbium; **E** embolus; **m29** muscle 29; **m30** muscle 30; **mA** median apophysis; **mH** median hematodocha; **PE** process of embolus; **S** spermophor; **St** subtegulum; **Te** tegulum; **tm29** tendon of muscle 29; **tm30** tendon of muscle 30.

(ZIMG II/28127; Fig. 5).

##### Male palp.

Tegulum with short median apophysis. Embolus tube-like with a solid single hook-shaped process; tip of the embolus widened and hyaline; fundus sac-like, spermophor with an internal band delimited by two parallel ridges (Fig. 5B), distal part of the spermophor within the embolus partly convoluted and very thin (approximately 8µm in diameter); m30 and tm30 in cymbium; m29 in tibia and attaching to the spermophor.

##### Natural history.

The web structure is similar to *Progradungula carraiensis* (for details see Gray 1983) and web construction is performed after sunset. The catching ladders and supporting webs (Figs 2B–C) are connected by a single sturdy thread to retreats in hollow trees Fig. 2A), often far away (up to 3 meters). As already described by Milledge (1997), we found most of the specimens in hollows of old *Nothofagus cunninghamii* trees (Fig. 2A), but also several in hollows of mountain ash (*Eucalyptus regnans*) trees, the bases of large tree ferns (Fig. 2B) and under bridges on trails. On one occasion, we had access to a large hollow mountain ashtree and found catching ladders and supporting webs of juveniles inside of it.

##### Distribution.

Endemic to the Great Otway National Park (Victoria). In addition to the locations reported by Milledge (1997), we found several specimens (one male, several juveniles) at the Melba Gully Trail which is located at the western end of the National Park (Fig. 1).

## Discussion

Our data are in accordance with the previous detailed descriptions on the distribution and natural history of *Progradungula* species (Forster and Gray 1979; Forster et al. 1987; Gray 1983; Milledge 1997). The new records of *Progradungula otwayensis* from the Melba Gully trail might imply a wider distribution in the Great Otway National Park. Nevertheless, this species seems to be dependent on hollow trees with a suitable microclimate (Gibbons and Lindenmayer 1997; Gibbons et al. 2002), since specimens mostly occur in the oldest and extensively hollow myrtle beech trees in the humid forests in the western part of the Great Otway National Park, or in mountain ash trees, upon which the myrtle trees depend (Lindenmayer et al. 2000). Other habitats such as tree ferns were much less inhabited in areas where old hollow myrtles occur (Milledge 1997). The presence of juvenile catching ladders and supporting webs inside the hollow trees suggests that early instars live exclusively in hollows. This is also supported by the fact that we could never observe exposed juvenile catching ladders, as we abundantly found for larger immatures and adults.

The female genitalia of *Progradungula otwayensis* are very similar to *Progradungula carraiensis* as the number of spermathecae is reduced compared to other gradungulids and they are bilaterally arranged (Forster et al. 1987). Thus, we here confirm the generic placement suggested by Milledge (1997). Additionally, we found distinct and sclerotized pockets in a fold anteriorly to the epigastric furrow which has not been reported from any other gradungulid (Forster et al. 1987). The function of these pockets is unknown, but an interaction with the embolic process during copulation seems to be a possibility. Moreover, in contrast to Forster et al. (1987) we could not find a “median receptaculum”. Instead, the organization is mainly in accordance with the interpretation of Griswold et al. (2005) of the genitalia of gradungulids.

As revealed by micro-CT analysis of the male palpal organ of *Progradungula otwayensis*, the spermophor has a thick wall and an internal band delimited by two parallel ridges, which in the micro-CT sections appears associated with glandular tissue. As described by Forster et al. (1987) for *Kaiya* Gray, 1987, the spermophor of *Progradungula otwayensis* is convoluted within the embolus. The muscles (m29 and m30) and their tendons are in accordance with the description of *Gradungula sorenseni* Forster, 1955 by Huber (1994) and resemble the usual organization for non-entelegyne Araneomorphae (Huber 2004). Thus, the organization might be typical for all Gradungulidae and different to the organization found in austrochilines (Michalik and Ramírez in press).

## Supplementary Material

XML Treatment for Progradungula otwayensis
